# Quantitative and Correlational Analysis of Brain and Spleen Immune Cellular Responses Following Cerebral Ischemia

**DOI:** 10.3389/fimmu.2021.617032

**Published:** 2021-06-08

**Authors:** Qingkun Liu, Siamak K. Sorooshyari

**Affiliations:** ^1^ Department of Neurology, School of Medicine, Stanford, CA, United States; ^2^ Icahn School of Medicine at Mount Sinai, New York, NY, United States; ^3^ Department of Integrative Biology, University of California, Berkeley, Berkeley, CA, United States

**Keywords:** cerebral stroke, brain, spleen, immune cells, function

## Abstract

Stroke is a multiphasic process, and the initial ischemic phase of neuronal damage is followed by secondary innate and adaptive responses that unfold over days after stroke, offer a longer time frame of intervention, and represent a novel therapeutic target. Therefore, revealing the distinct functions of immune cells in both brain and periphery is important for identification of immunotherapeutic targets for stroke to extend the treatment time window. In this paper an examination of the cellular dynamics of the immune response in the central nervous system (CNS) and periphery provoked by cerebral ischemia is provided. New data is presented for the number of immune cells in brain and spleen of mice during the 7 days following middle cerebral artery occlusion (MCAO). A novel analysis of the correlation among various cell types in the brain and spleen following stroke is presented. It is found that the infiltrated macrophages in the ischemic hemisphere positively correlate with neutrophils which implies their synergic effect in migrating into the brain after stroke onset. It is noted that during infiltration of adaptive immune cells, the number of neutrophils correlate positively with T cells, which suggests neutrophils contribute to T cell infiltration in the stroked brain. Furthermore, the correlation among neurological deficit and various immune cells suggests that microglia and splenic adaptive immune cells (T and B cells) are protective while infiltrating peripheral myeloid cells (macrophage and neutrophils) worsen stroke outcome. Comprehension of such immune responses post cerebral ischemia is crucial for differentiating the drivers of outcomes and also predicting the stroke outcome.

## Introduction

**Figure 1:**
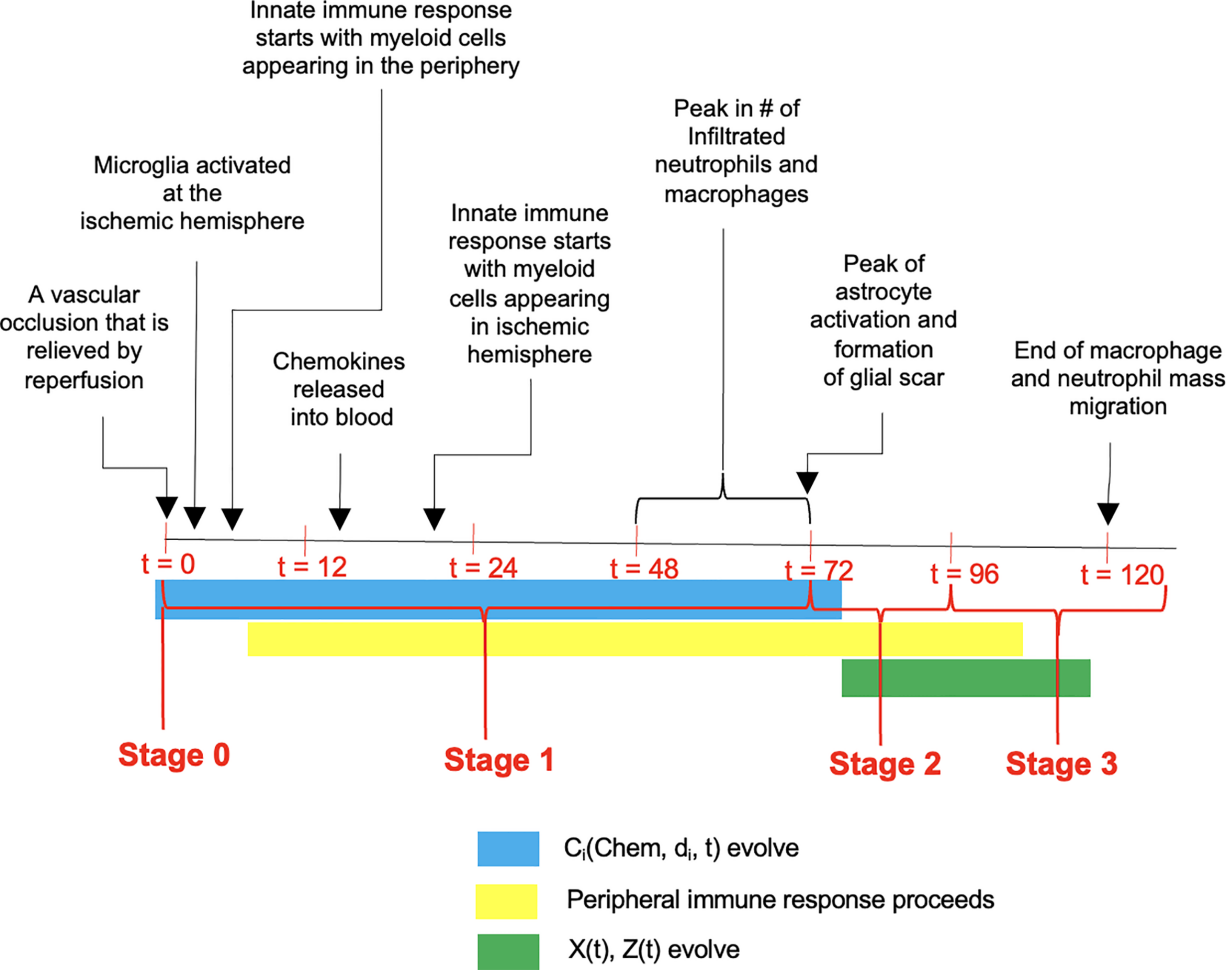
A time-line of events that unfold following the ischemic insult. The progression has been divided into three stages with Stage 0 representing the occlusion, and Stage 1 encompassing the response of the CNS and peripheral immune systems including reduction in microglial population and migration of peripheral innate immune cells into the CNS. Stage 2 is characterized by plateau phase of microglia and infiltrated myeloid cells, coupled with continuous infiltration of peripheral adaptive immune cells into the CNS. During the stage 3 (i.e., between 96h and 120h post stroke), the infiltrated myeloid cells gradually diminish and vanish, while microglial population return back to homeostasis accompanied by persistent presence of adaptive immune cells in the CNS.

**Figure 2:**
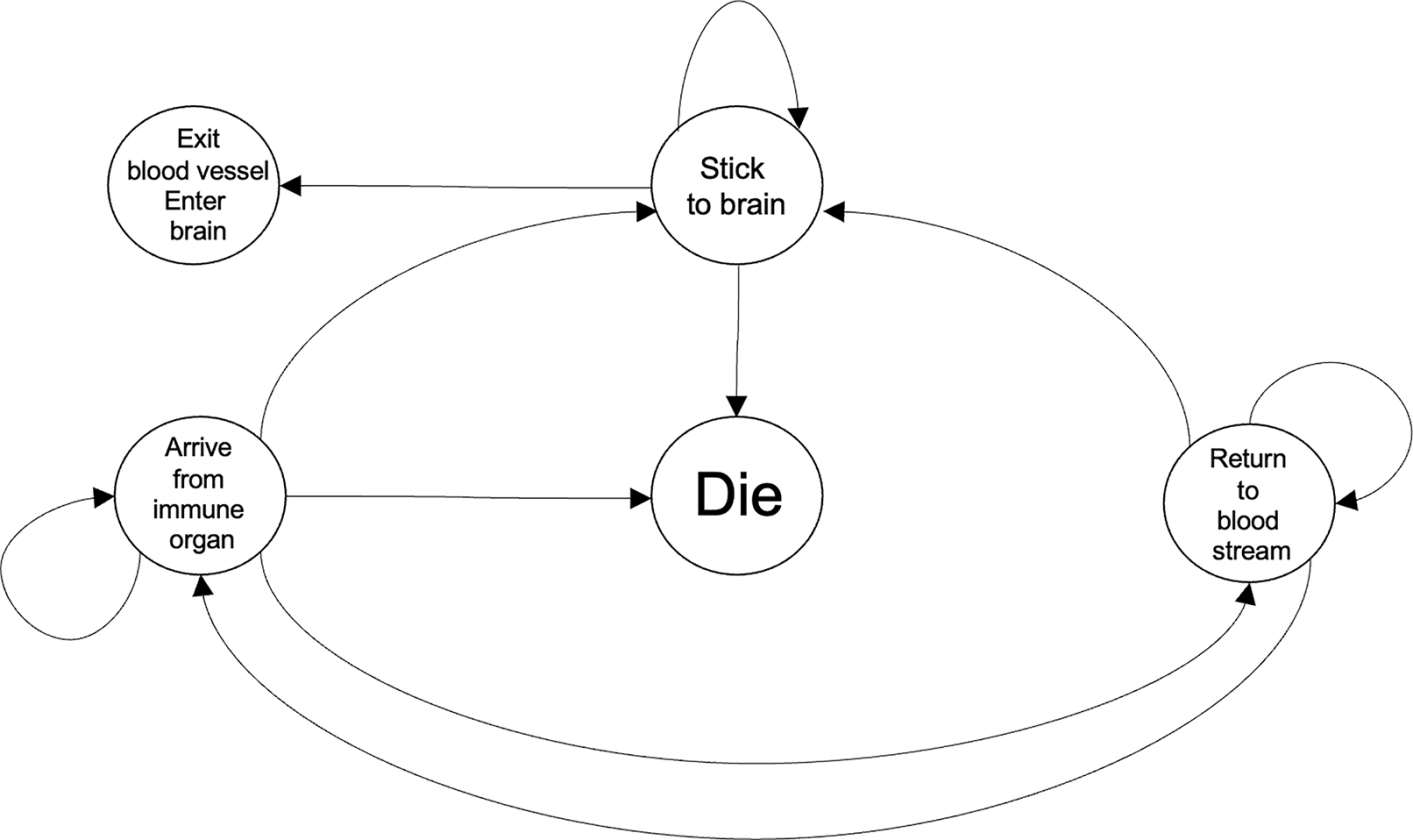
Representative dynamics of the scenarios encountered by an immune cell while coming to the ischemic brain in response to chemokines and DAMPs. The Die and the Exit blood vessel, Enter brain states refer to what would take place at the blood vessels and the brain, respectively. Within a computational framework, the two aforementioned states would be referred to as absorbing states because once an immune cell enters either of those conditions it will not leave that state. Each transition can be associated to a transition probability with the system approximated by a Markov chain. It would be expected that the state transition probabilities will be time-varying and also dependent on a common set of biological variables.

**Figure 3:**
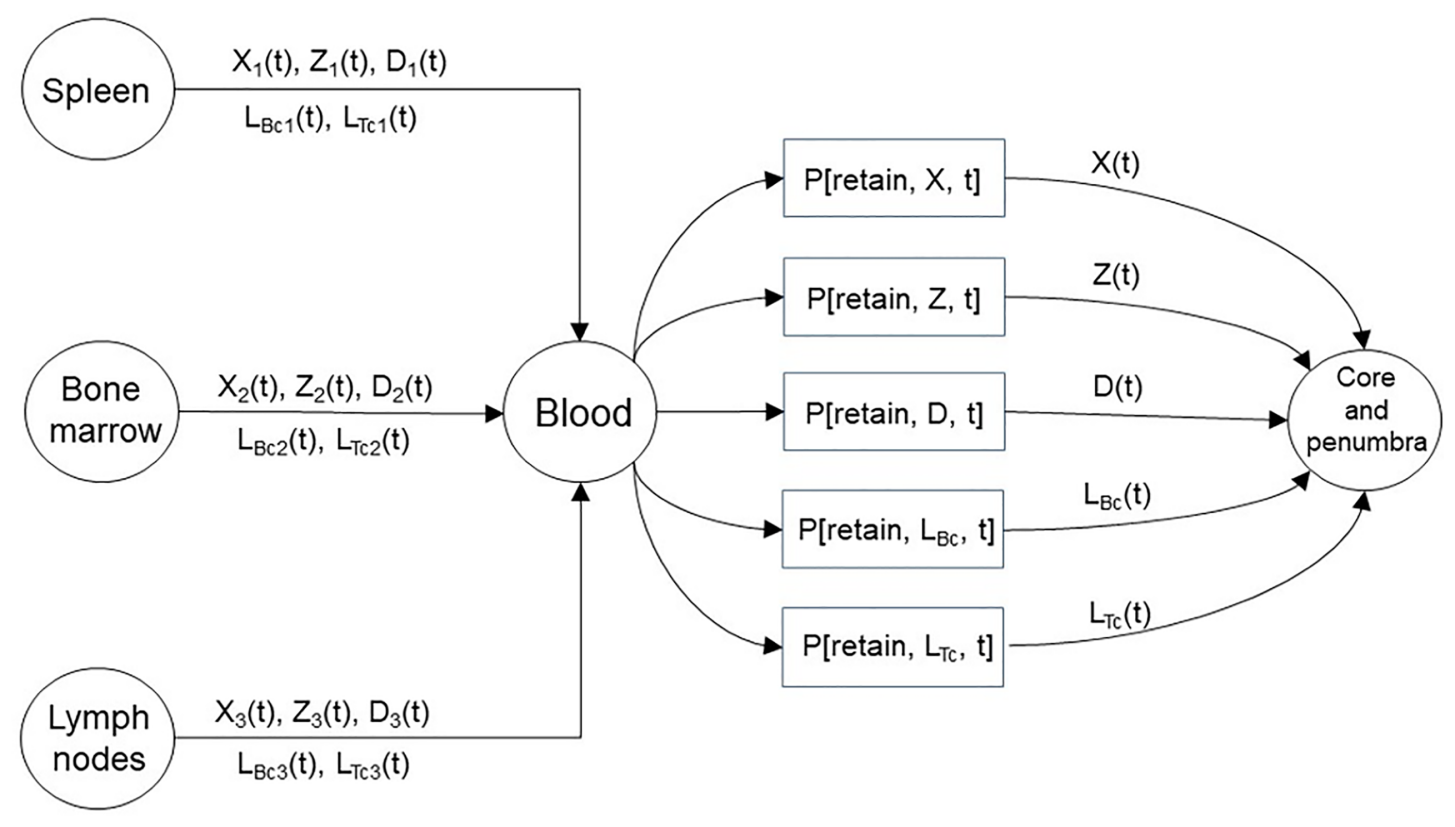
A representation of the immune organs that provide the macrophages, neutrophils, dendritic cells, and lymphocytes that infiltrate the core and penumbra following stroke. The immune organs shown represent three distinct sources with the brain being the destination. The amount of immune cells traversing from the spleen, bone marrow, and lymph nodes are written above and below the medium connecting the source-destination pairs. The cellular travel illustrated above occurs as a consequence of a chemokine gradient. The presented model introduces the as time-varying quantities that contribute to the formation of the infarct by regulating the retention of the emitted immune cells by the three sources through the blood.

Ischemic stroke is the fourth leading cause of death and the leading cause of disability in the United States. The time window for vascular intervention after stroke, using either thrombolytic therapy or endovascular surgery, is limited and neuroprotective approaches have failed in clinical trials. On the other hand, stroke is a multiphasic process with the progression of ischemic brain injury associated with intense and long-lasting innate and adaptive immune responses (1, 2) that are biologically distinct, offer a time frame for intervention, and represent a novel therapeutic target. There is evidence that immune responses following the onset of stroke is not only a consequence of the injury – but can also contribute to secondary brain damage as well as provide beneficial effects. However, the dynamics and functions of the immune cells that drive cerebral injury have yet to be clearly identified despite various experimental manipulations (for example, splenectomy) having shown proof of principle that components of the post-stroke immune response are injurious. Knowledge of the dynamics and functions of immune cells after stroke will provide insight crucial to the development of therapeutic interventions to improve outcome. **Figure 1** is a time-line of the central nervous system (CNS) and peripheral immune system responses following cerebral ischemia and will be referred to as the events that are expected to occur while considering data during the shown time points. From a computational perspective, post-stroke immune response is a complex system where the constituent processes are correlated and have beneficial as well as deleterious effects. The systemic nature of stroke points to the necessity of accounting for cell dynamics from multiple systems such as the central nervous and immune systems (3). In this work, attention is restricted to the dynamics of and correlation among brain and splenic immune cells. More specifically, we present analysis for post-ischemic CNS and splenic immune responses based on our data from the mouse stroke model of transient middle cerebral artery occlusion (MCAO). At the blood-brain barrier (BBB), the interactions between the physiological states that an immune cell encounters upon arrival are illustrated in **Figure 2**. It is posited that the presented analysis is general enough to be applicable to other animal models and eventually provide insight into the evolution of clinical stroke. A high-level quantitative view of the ischemic process is illustrated *via* the schematic in **Figure 3** and data is presented for the number of immune cells in the brain and spleen at different intervals between 1 and 7 days following MCAO. The dynamics of cell accumulation in the contralateral (non-stroked) and ipsilateral (ischemic) hemispheres are compared with those reported by Gelderblom et al. (2). Subsequently we present a correlational analysis of the dynamics of the various cell types by computing the Pearson correlation among the number of cells that have been measured across different animals at various time points during the post-stroke immune response. The findings are important in elucidating relationships among the different classes of cells, differences among the same cell type in the stroke versus sham condition, and relations between the cell types present in the brain and the spleen.

From a translational perspective, several metrics have been used to quantify the harmful effects of cerebral ischemia. The degree of ensuing weight loss has been viewed as a useful measure, as have the infarct volume and neurobehavioral outcomes including the loss of motor function. In this work the neurological deficit score (NDS) (4) is measured and used as the determinant of stroke outcome. It is important to correlate NDS with the various cell counts in the brain and spleen with the aim of discovering whether a change in cell number at a particular time after stroke may be predictive of the outcome. In fact, a promising avenue in stroke research will be to predict outcome from the dynamics of various cell types throughout the body. A step in this direction is provided here *via* the data and analysis.

## Methods

### Animals

This study was conducted in accordance with the National Institutes of Health (NIH) guidelines for the use of experimental animals. Animal protocols were approved by the Institutional Animal Care and Use Committee (IACUC). Wild-type C57BL/6 male mice were purchased from Jackson Laboratories (JAX; Bar Harbor, Maine, USA). All mice were housed in an environment controlled for lighting (12-hour light/dark cycle), temperature, and humidity, with food and water available ad libitum.

### Transient Focal Ischemia Model

Mice of 8-12 weeks were randomized and subjected to sham surgery or 45 minutes of occlusion of middle cerebral artery (MCA) followed by reperfusion, with survival out to 7 days maximum. Transient middle cerebral artery occlusion-reperfusion (tMCAo-RP) experiments were performed as described previously (5, 6). In brief, animals were anesthetized with isoflurane (4% induction and 2.0% maintenance), after anesthetic induction the animals were placed on a heating pad to maintain the body temperature at 36.8–37.2°C during the surgical procedure. To induce the occlusion of MCA, a monofilament of diameter 0.15 to 0.18 mm was inserted into external carotid artery (ECA) and reached the common carotid artery (CCA) junction. The filament was then inserted into the internal carotid artery (ICA) which was up to 9-11 mm into the MCA. After confirmation of MCA blockage, the filament was withdrawn following 45 minutes of occlusion. The mortality ratio of stroke mice was 19.15% at day 1 and 25.97% at day 2 post stroke. The occlusion and reperfusion of the MCA is confirmed in mice by dissection of the stroked brain and measurement of the NDS. In particular, animals with hemorrhage or without infarct in the ipsilateral hemisphere were excluded. Animals showing an NDS less than 1 at 3h post-surgery are also excluded. Assessment of the NDS on the mice were performed and scored as follows: 0, no deficit; 1, forelimb weakness and torso turning to the ipsilateral side when held by the tail; 2, circling to affected side; 3, unable to bear weight on affected side; and 4, no spontaneous locomotor activity or barrel rolling (7).

### Isolation of Brain and Splenic Immune Cells for Flow Cytometry

Mice were anesthetized with isoflurane and then transcardially perfused with 0.9% saline (wt/vol), followed by collection of brain and spleen samples. Spleen samples were homogenized in 10ml 0.01 M PBS and filtered through a 70 μm cell strainer to isolate single cells. These cells were then concentrated by centrifugation as pellets followed by incubation in 3ml RBC lysis buffer (1:10, Biolegend, USA) for 5 min on ice to lysis the red blood cells. The lysed red blood cells were then eliminated *via* centrifuge as supernatant. Splenocytes were re-suspended in 200 µl 0.01MPBS for flow cytometry. Brain hemispheres were homogenized in 10ml 1x Hank’s balanced salt solution (1xHBSS, Thermo Fisher, Waltham, USA), and filtered through a 70µm cell strainer. After wash with 0.01M PBS, myelin digestion was then carried out with collagenase IV (1mg/ml in HBSS, Worthington, Lakewood, NJ) incubation for 30 min at 37°C and shaking at 200 rpm. Myelin was separated by density gradient centrifugation (1500xg for 7 min) in 30% Percoll (vol/vol in 0.01 M sodium-potassium PBS, Sigma, St Louis, USA). Cells were resuspended in 200ul PBS for flow cytometry. Dead cells were identified using LIVE/DEAD^®^ Fixable Dead Cell Stain Kit (Thermo Fisher Scientific, USA) according to the manufacturer’s protocol. Cells were fixed with 1.6% paraformaldehyde (PFA in 1xPBS) for 10min at room temperature and stored at -80°C for flow cytometry.

### Flow Cytometry Analysis of Brain and Splenic Immune Cells

**Figure 4:**
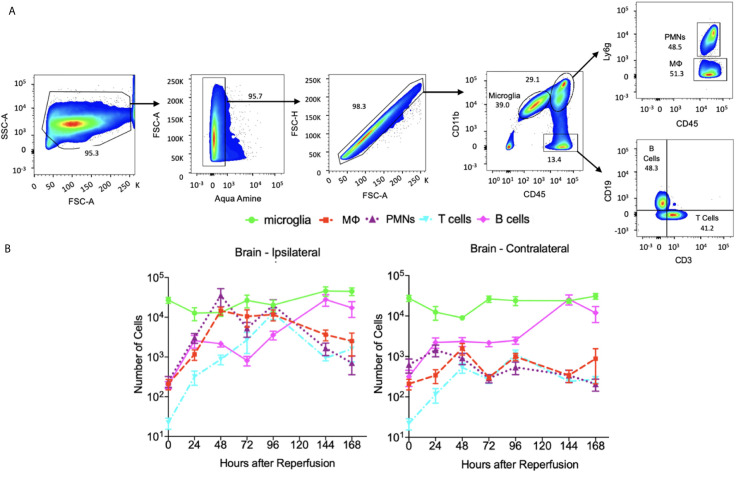
The dynamics of immune cells at ipsilateral and contralateralhemispheres post stroke and sham. **(A)** flow cytometry plots showing the gate strategy for identification of microglia, neutrophil (PMNs), macrophage (MΦ), T cells and B cells. In brief, alive singlet cells from brain were chosen for analysis on multiple biomarkers expression level. In particular, microglia were identified as CD45^+^CD11bMed, neutrophils as CD45+CD11b^hi^Ly6G^+^, macrophage as CD45^+^CD11b^hi^Ly6G-, T cells as CD45^+^CD11b-CD3^+^CD19- and B cells as CD45^+^CD11b-CD3^+^CD19-. **(B)** A log-scale figure shows the number of various immune cells present in brain until 7 days post stroke. N=7-10 mice were used across the 7 days of analysis post stroke.

**Figure 5:**
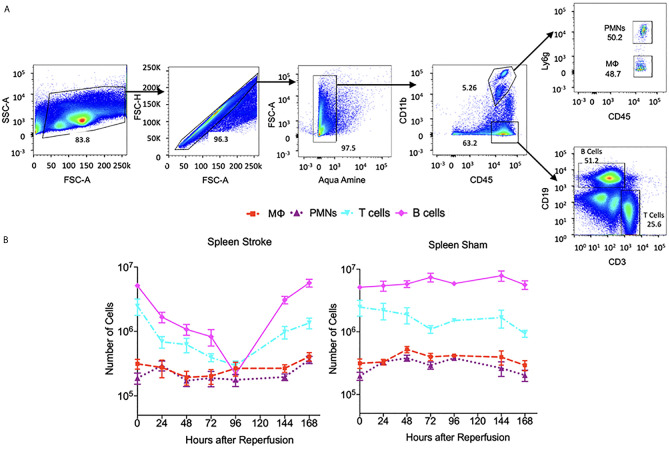
The dynamics of immune cells at the spleen post stroke and sham. **(A)** Flow cytometry plots showing the gate strategy for identification of neutrophil (PMNs), macrophage (MФ), T cells and B cells. In brief, alive single cells from spleen were chosen for analysis on multiple biomarkers expression level. In particular, neutrophils were identified as CD45^+^CD11b^hi^Ly6G^+^, macrophage as CD45^+^CD11b^hi^Ly6G ,T cells as CD45^+^CD11b-CD3^+^CD19-and B cells as CD45^+^CD11b-CD3^+^CD19-. **(B)** A log-scale figure shows the number of various immune cells present in spleen until 7 days post stroke. N=7-14 were used across the 7 days of analysis post stroke or sham.

All samples were acquired as described above. ~10^6^ cells were suspended in 200 μl HBSS buffer and incubated with anti-mouse CD16/32 (5 ng/μl, BioLegend, clone 93) for 10 min at 4°C to block Fc-receptor binding. Dead cells were discriminated using Aqua Amine according to the manufacturer’s protocol (LIVE/DEAD^®^ Fixable Dead Cell Stain Kit, Thermo Fisher Scientific, Waltham, MA). The following antibodies were used for surface receptor detection to identify neutrophil, macrophage, microglia, T cells and B cells: CD45 (1 ng/μl, Biolegend, clone 30F-11), CD11b (1 ng/μl, Biolegend, clone M1/70), Ly6G (1 ng/μl, Biolegend, clone 1A8), CD3 (1ng/ul, Biolegend, clone17A2) and CD19 (1ng/ul, Thermo Fisher, clone SJ25-C1). Stained Cells were then washed with HBSS buffer, resuspended in 200 μl of HBSS and analyzed with a LSRII cytometer (BD biosciences, San Jose, CA) and FlowJo software (Tree Star Inc, Ashland, OR). In particular, neutrophil is identified in the **Figures 4** and **5** flow cytometry plots as CD45^hi^CD11b^+^Ly6g^+^, macrophage as CD45^hi^CD11b^+^Ly6g^-^, microglia as CD45^med^CD11b^+^, T cells as CD45^+^CD11b^-^CD3^+^CD19^-^ and B cells as CD45^+^CD11b^-^CD3^-^CD19^+^.

### A Three-Stage Account of Immune Cell Accumulation

We consider three successive temporal intervals as being reflective of the cellular phenomena that follow the insult and discuss quantifiable parameters for each stage. Due to the lack of astrocyte and neuron data, we restrict attention to macrophages, microglia, neutrophils, dendritic cells, and lymphocytes. A description of the three stages is provided as we consider the temporal progression of the immune response following MCAO in mice. A primary aim of the analysis is to elucidate the dynamics of immune cells that travel to the brain and spleen following the insult. As with virtually any biological modeling effort, the presented analysis will be a simplification and not a comprehensive account of the underlying reality. For instance, cell count changes due to *in situ* apoptosis and proliferation have not been considered. The variables that shall be used throughout this work to refer to various processes are listed in **Table 1**. 

**Table 1:** A litany of the variables discussed as being important in the inflammatory response following stroke and used to quantify the processes considered in this work.

Variable	Description
X(t)	The number of macrophages. The cells are differentiated into distinct groups X_M1_(t) and X_M2_(t) to denote the number of macrophages expressing the toxic (M1) and beneficial (M2) phenotypes, respectively. A subscript of i=1, 2, 3 will denote the spleen, bone marrow, or lymph nodes, respectively, being the source of the cell.
Y(t)	The number of microglia cells. The microglia expressing the M1 (M2) phenotype will be designated as Y_M1_(t) (Y_M2_(t)) to designate their toxic (beneficial) function.
Z(t)	The number of neutrophil cells. A subscript of i=1, 2, 3 will denote the spleen, bone marrow, or lymph nodes, respectively, being the source of the cell.
D(t)	The number of dendritic cells. Monocytes differentiate into macrophages and dendritic cells. A subscript of i=1, 2, 3 will denote the spleen, bone marrow, or lymph nodes, respectively, being the source of the cell.
L_Bc_(t), L_Tc_(t)	The number of lymphocytes. We consider B cells and T cells with quantities denoted by L_Bc_(t) and L_Tc_(t), respectively. An additional subscript of i=1, 2, 3 will denote the spleen, bone marrow, or lymph nodes, respectively, being the source of the cell.
P[retain, ., t]	The retention probability, defined as the probability of a cell type adhering to the infarct following stroke.

It is largely accepted that following the initial insult, macrophages and neutrophils travel to the area of infarction from blood, lymph nodes, spleen, and bone marrow *via* the blood stream. Neutrophils are believed to arrive later than the macrophages, approximately 24 hours after the insult, and contribute to injury (2, 8). Accumulation of neutrophils has been correlated with an increase in the severity of brain tissue damage and poor neurological outcome (9, 10). The resident brain macrophages, or microglia, are embedded in the brain parenchyma and thus are among the first cells to respond. Microglia that survive the ischemic episode are activated in the brain and there is a range of phenotypes associated with microglial activation following ischemia. The M1 phenotype – generally considered pro-inflammatory – release primarily destructive pro-inflammatory mediators and reactive oxygen species (ROS). The M2 phenotype – generally believed to be anti-inflammatory – release anti-inflammatory cytokines that contribute to repair or neuroprotection. An important caveat is that the effects of the M1 and M2 microglial phenotypes are not exclusive (i.e. there is a degree of heterogeneity and overlap) with each containing several subsets of phenotypes. In presenting the analysis and dynamics, the microglia were not divided into M1 or M2 subpopulations but rather considered as one population.

### A Derivation of Quantitative Relationships for the Arrival of Immune Cells at the Core and Penumbra

Cytokines and ROS from activated microglia or damaged neurons induce the expression of cellular adhesion molecules by the endothelial cells at the BBB. The traveling immune cells which themselves express complementary adhesion molecules will adhere to the vasculature and may enter the brain tissue (11). During the post-stroke inflammatory response, immune cells can undergo one of the following: they may be released from a peripheral immune organ and retained in the brain, return to the blood stream prior to being remitted back to the periphery, traffic into the parenchyma and later migrate into the area of infarct, or die after not reaching the immune organs and not having undergone adhesion to remain in the area of infarct. In the final scenario, following death, the cell may be phagocytosed in-place. From a quantitative perspective, for each immune cell type shown in **Figure 3** a probability will multiply the number of released cells to provide a tally of the amount that remain in the area of infarction and become part of the infarct through adhesion followed by diapedesis (i.e. leukocyte migration into the brain parenchyma). In effect, it is possible to model the stages of cell adhesion and blood vessel escape in unison. In **Figure 3** the index i = 1, 2, 3 denotes the various cell types considered in this work being released from the spleen, bone marrow, and lymph nodes, respectively. We refer to the probability of a cell adhering to the infarct as a retention probability, and present the relations

(1) $$\begin{array}{c} X(t)=P[retain,X,t](X_{1}(t)+X_{2}(t)+X_{3}(t))\\ Z(t)=P[retain,Z,t](Z_{1}(t)+Z_{2}(t)+Z_{3}(t))\\ D(t)=P[retain,D,t](D_{1}(t)+D_{2}(t)+D_{3}(t))\\ L_{Tc}(t)=P[retain,L_{Tc},t](L_{Tc1}(t)+L_{Tc2}(t)+L_{Tc3}(t))\\ L_{Bc}(t)=P[retain,L_{Bc},t](L_{Bc1}(t)+L_{Bc2}(t)+L_{Bc3}(t)) \end{array}$$

to represent the evolution of the number of released immune cells by the three organs and the portion that becomes part of the infarct. The retention probabilities are determined largely by the adhesion molecules at the BBB, and are thus dependent on the time after stroke onset and the cell type. The immune cells on the right-hand-side of the equalities in (1) are present in the peripheral blood post-MCAO; whereas the immune cells on the left-hand-side are in the brain post-MCAO. With the suggested relations in (1) and prospective data, one could calculate estimates for the retention probabilities. Ultimately, having reliable estimates of the retention probabilities, and a measurement of the number of immune cells in the peripheral blood would allow one to predict the number of immune cells that adhere to the area of infarct, predict the infarct volume, and potentially infer the stroke outcome.

### Statistical Dependence Among Cell Types and the Neural Deficit Score

The Pearson correlation (via corrcoef in MATLAB R2020a) was used to assess the prospective statistical dependence among the dynamics of the various cell types in the brain and spleen as well as the statistical dependence among the number of cells and the NDS. The number of animals used in computing the mean of a cell type for a particular day ranges from 5 to 14 (see **Supplementary Table 1** for number of animals where cell counts were taken at each day). This range of samples is because outlier removal was performed when examining the number of the various cell types measured over the days for each animal. The Prism software’s ROUT method was used with the maximum desired false discovery rate (FDR) set to 1%. The outliers stemmed from either biological or technical reasons such as individual variations or experimental operation due to the large number of animals used. When assessing the correlation between various cell types present in the brain or spleen, each cell type was averaged across all the animals for a particular day. We considered the entire 7 day interval as well as the subintervals of 0-3 days and 4-7 days post MCAO. The three intervals corresponded to Pearson correlation coefficients being computed among two mean vectors of lengths 8, 4, and 4, respectively. In calculating the correlation among NDS scores and the number of cells in the brain or spleen; the mean NDS was computed across N=14 mice for each day. The resultant vector was correlated with a vector of mean cell counts computed across the available number of animals for each day.

## Results

The dynamics and time course of the influx of various cell types to the brain and immune organs during the formation of the infarct are discussed *via* three-stages. The stages have been identified in chronological order following the insult, and a general illustration of the events implicated during the stages is presented in **Figure 1**. Analysis of the number of cells present during the stages will reveal differences between stroke and sham conditions. Furthermore, a study of the correlation among the different cell types will elucidate whether the dynamics of one cell type is reflective of the dynamics of another cell type, and whether the arrival of a cell at the brain is reflective of the arrival of that cell type at the spleen. From a quantitative perspective this is a step in understanding the dependencies among the cellular processes that are conceived to influence stroke outcome. By having the capability to correlate such factors with the NDS, it will be possible to delineate cell dynamics that are biomarkers of stroke outcome.

### A Three-Stage Description for Cellular Dynamics Following Cerebral Ischemia

#### Stage 0: The Ischemic Insult

The initial stage will consist of a vascular occlusion that is relieved by reperfusion at time t=0. Several factors at this stage can affect the subsequent inflammatory response and its pathological consequences. An increase in duration of occlusion leads to longer ischemia and increases infarct size, leading to greater release of cytokines and a greater influx of the immune cells. Furthermore, elongated occlusion may permit the released cytokines to surpass an effective signaling concentration at a peripheral site. A larger infarct was noted with the higher occlusion times as we considered the length of occlusion (LOO) of 30, 45, and 60 minutes (**Supplementary Figure 1**). The increase in infarct size between the 30-minute LOO and the two longer scenarios were statistically significant, while the change between the 45-minute and 60-minute cases were not significant. The effects of an MCAO-induced stroke are initially observed in the striatum due to its proximity to the occluded MCA. If the LOO is extended, then the effects are seen in more distal vascular beds, namely in the cerebral cortex (12). Factors known to influence stroke outcome that are not considered in the analysis due to insufficient data include collateral circulation, spatial location of the stroke, and gender.

#### Stage 1: Formation of the Infarct Volume and Infiltration of Peripheral Immune Cells

We consider the interval 0 < t ≤ 72 hours as the acute stage immediately following reperfusion. The earliest part of this stage contains the juncture 0 < t < 12 hours when the infiltration of myeloid cells start, microglia become activated, and cytokines are released from the brain (2). Reperfusion is typically followed by a surge in the production of radicals and ROS such as superoxide, nitric oxide, and peroxynitrate. Formation of these radicals in the vicinity of blood vessels plays an important role in reperfusion-induced injury by increasing the BBB permeability. The oxidative and nitrative stress also promote the recruitment of neutrophils and other leukocytes to the cerebral vasculature. While low levels of free radicals are known to perform signaling functions and are believed to be benign, high levels are detrimental and contribute to increasing the infarct volume. Concurrently, the peripheral immune response is a continuous process which commences within hours of stroke onset, typically before the 12-hour-point. This is a process that extends into and beyond Stage 2 as described in the ensuing sections and entails the activation of macrophages, neutrophils, dendritic cells, and T and B lymphocytes. The number of the aforementioned cells have been shown to be relatively constant across MCAO trials in mice (2, 13). Commencing at Stage 1 and continuing throughout the ischemic process, dying brain cells contribute to inflammation by releasing cytokines and damage associated molecular pattern molecules (DAMPs). The cytokine release is either a direct cause or a secondary effect of the release of DAMPs. Concurrently, there is evidence that microglia are a major source of cytokine release following stroke (14).

By definition, the core is the region that is most severely deprived of blood flow and thus all cell types, including the resident microglia, within the core will die. In the surrounding penumbra the decrease in blood flow is less pronounced, does not affect all of the encompassed cells, and cell death occurs over a longer time scale. The surviving microglia become activated and start proliferating until plateauing at around the t=24 hour mark (2). Upon activation, the microglia clear dead cells and release cytokines. In fact, the number of microglia of phenotype M1, M2, and their variants are believed to increase with time during Stage 1. Spatially, the microglia reside in the CNS and undergo activation *in situ* whereas the macrophages require time to arrive from different locations. Macrophages arrive at the area of infarction within the first two days after the activation of the microglia (15, 16). Throughout this stage the macrophages are exposed to damaged cells as well as released cytokines/chemokines, and begin to assume different activated phenotypes during their travel. An essential function of the microglia and macrophages is to clear cellular debris by phagocytosis, and also contribute to the initiation of the adaptive immune process that is implicated as a contributor to plasticity during post-stroke recovery (17). The initiation includes the presentation of antigens and signaling to induce the travel of T and B cells to the brain. The conclusion of this stage at t=72 hours is distinguished by the peak of astrocyte activation inducing the later formation of a glial scar.

#### Stage 2: Early Evolution of the Infarct and Plateauing of the Infiltrated Myeloid Cells

The period of 12 ≤ t ≤ 96 hours encompasses a continuous migration of peripheral myeloid cells from the periphery, including the spleen, to the brain. Similar to microglia, macrophages may be polarized to either a pro-inflammatory (M1) or an anti-inflammatory (M2) phenotype while recognizing that each contains multiple sub-phenotypes. Importantly, macrophages of either M1 or M2 phenotype can perform phagocytosis, and there are two general functions that are not exclusive to the phenotype. The first function is deemed beneficial and consists of clearing debris and dead tissue as well as releasing trophic factors that help neurons survive during the ischemic response. The second function is deemed deleterious and consists of release of toxic cytokines and free radicals.

Over the initial 96 hours post-stroke, immune cells in the spleen undergo cell death as well as cell division and migration out of the spleen. The spleen shrinks during this stage with higher levels of shrinkage typically corresponding to a larger infarct volume. The infiltration of the macrophages, neutrophils, and dendritic cells into the ischemic brain has been shown to precede that of the lymphocytes (18–22). The numbers of macrophages and neutrophils decline over the first 96 hours following the insult (15).

#### Stage 3: Progression of the Infarct and Termination of Myeloid Cell Infiltration

The number of myeloid cells in the spleen, initially decline over the first 2-4 days after the ischemic event, begin to recover after 96 hours and normalize by day 7 post stroke (15) as shown in the cell count dynamics in **Figure 5**. Although permanent injury to the core of the infarct is likely to be complete by the end of Stage 1, the response of the penumbra to prior ischemia is an ongoing process that extends into the later recovery phase. This may be reflected in the changes that microglia undergo across the progression of the ischemic brain injury. The M2 phenotype microglia steadily increase until approximately t = 120 hours (5 days) at which point they start to decrease until being virtually non-existent 14-days after the stroke. Conversely, the M1 phenotype microglia continue their course of increase to this point (13).

### Microglia Show Similar Dynamics Among the Ipsilateral and Contralateral Hemispheres After Stroke

**Figure 6:**
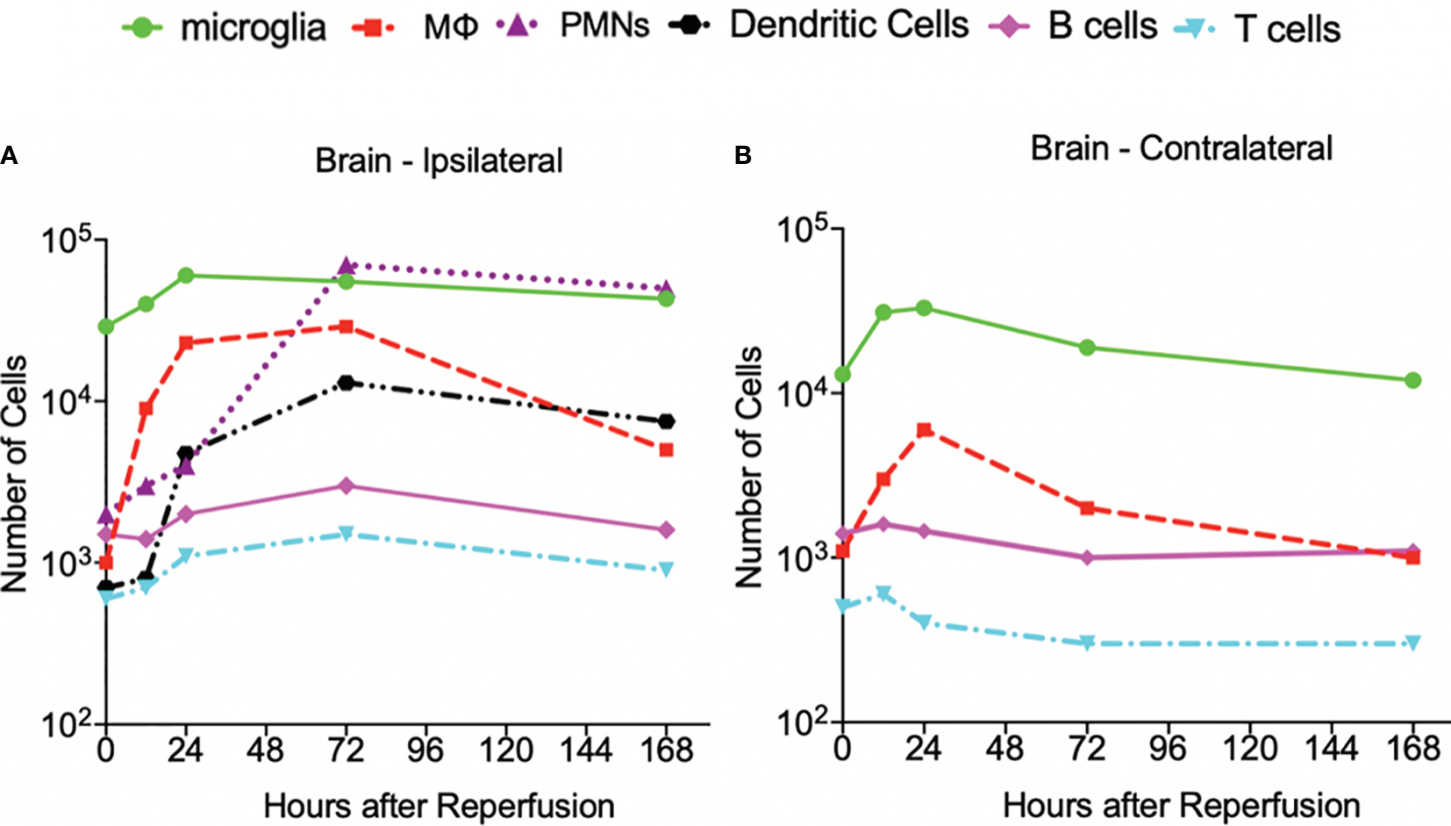
The dynamics of the cell count as a function of hours after insult obtained from the data presented in reference (2). **(A)** At the ipsilateral hemisphere of the brain. **(B)** At the contralateral hemisphere of the brain. A log­ scale is used to illustrate the vastly different number of cells on the same axes. N=3-4 mice were used for each time point and the experimented were replicated 3 times.

The dynamics of the number of microglia in the cortex and striatum appears similar for the M1 and M2 phenotypes (13). A finding of the aforementioned work is that the M1 phenotype microglia increase nearly monotonically over the 14-day recording period in the cortex and the striatum. In contrast, the number of M2 phenotype microglia peak at 5-days post-MCAO. We consider the data presented in (2) in **Figure 6** to note that the number of microglia in the ipsilateral and contralateral hemispheres are also similar since they peak at 24 hours post-insult prior to stabilizing by the 168-hour point. With respect to the data collected herein, the cell count dynamics plotted in **Figure 4**, further corroborate that the number of microglia are similar between the ipsilateral and contralateral hemisphere over the considered temporal window. However, **Figure 6** shows the number of microglia in both hemispheres plateauing much later than the 24-hour point.

### Dynamics of Infiltrated Immune Cells and Microglia in the Stroked Brain

For the data presented in Gelderblom et al. (2), the authors determined the number of macrophages and dendritic cells stained with CD40, CD80, and MHCII antibodies. The number of neutrophils were identified *via* high expression levels of CD45, and the amount of T-cell activation was identified *via* expression of the CD25 and CD69 markers. **Figure 6** indicates that the macrophage cell count dynamics in (2) are similar in both hemispheres although the number of cells was generally higher in the ipsilateral hemisphere. The macrophages reach their peak value relatively early after the insult – i.e. 24 hours in the contralateral and 72 hours in the ipsilateral hemispheres. Similarly, for the data collected herein, the cell dynamics shown in **Figure 4** indicate that there are more macrophages in the ipsilateral hemisphere at all measured time points. The number of macrophages peak at the 48-hour post-stroke time point in both hemispheres.

The data from (2) indicates that the number of B cells peaks later in the ipsilateral hemisphere (i.e. at 72 hours post insult) than the contralateral hemisphere. Aside from this, the B cell dynamics and cell counts do not seem to differ significantly when comparing the response at the two hemispheres. A similar finding is noted from the data collected herein as **Figure 4** indicates that the dynamics and number of B cells after stroke are similar across the ipsilateral and contralateral hemispheres. **Figure 6** indicates that the temporal dynamics of the number of T cells present in the two hemispheres are quite different for the data in (2). Namely, the number of T cells in the contralateral hemisphere peak at a much earlier point (i.e. 12 hours) than those in the ipsilateral (i.e. 72 hours). Furthermore, the number of T cells in the ipsilateral hemisphere is generally higher than that present in the contralateral hemisphere. Similarly, **Figure 4** indicates that for the data collected herein, after 24-hours post-insult, the number of T cells present in the ipsilateral hemisphere is much higher than that of the contralateral hemisphere.

While no contralateral neutrophil data was provided in (2), **Figure 6** shows that the number of this cell type in the ipsilateral hemisphere peaks at 72 hours post insult. Our data reveal that the number of neutrophils present in the ipsilateral hemisphere peaks earlier at 48 hours post insult, and in the contralateral hemisphere the number peaks at 24 hours post insult. Furthermore, the cell count dynamics in **Figure 4** indicate that after 24 hours, there are more neutrophils present in the ipsilateral hemisphere than the contralateral hemisphere.

### Dynamics of Immune Cells in the Spleen Following Stroke

The spleen has been implicated as a primary source of peripheral immune cells infiltrating into the ischemic brain. The change in spleen size after ischemic injury and its negative association with infarct volume are likely linked (23, 24) as suggested by studies where removal of the spleen was protective in stroke (25). The comparative plots in **Figure 5** indicate that in a sham spleen the number of T cells and B cells is much less variable over the 168 hours in comparison to that in a stroke spleen (a variance reduction of 46.7% and 76.3%, respectively). The sham spleen contains nearly an order of magnitude more B and T cells than macrophages and neutrophils over this time interval. The same phenomenon is not noted for a stroke spleen as the number of B and T cells drop rather precipitously at 96 hours post stroke prior to returning to their value at stroke onset (i.e. the 0-hour point). The number of macrophages and neutrophils in the stroke and sham spleen remain relatively constant during the 168 hour interval.

### Different Correlations Are Noted Among Immune Cells in Brain and Spleen After Stroke

**Figure 7:**
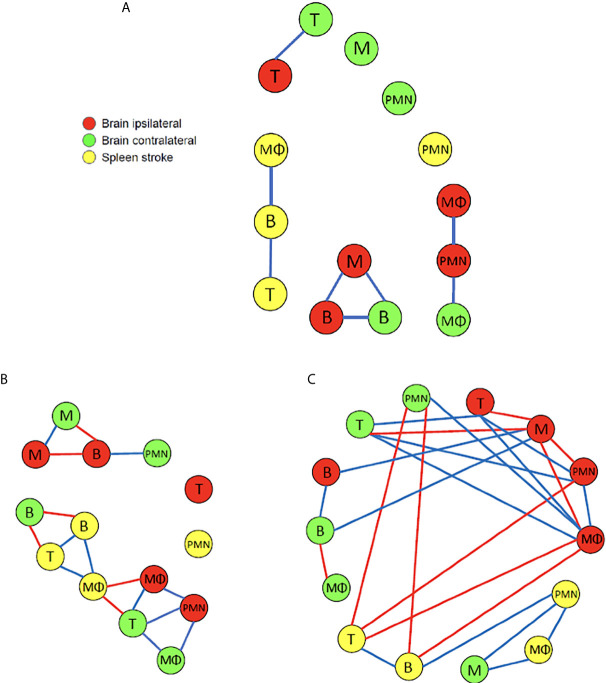
A graphical depiction of the cell types with dynamics that are highly correlated over time following the MCAO. The blue and red edges between nodes denote cell types that share high positive or negative correlation, respectively. **(A)** During the interval of day 0 to day 7 post insult a Pearson correlation coefficient value of |*ρ*| > 0.8 was deemed high (N=5-14). Interestingly, no high negative correlations are noted, and there is no high correlation among any of the cell types in the brain with those in the spleen. **(B)** During the interval of day 0 to day 3 post insult a Pearson correlation coefficient value of |*ρ*| > 0. 8 was deemed high (N=5-14). **(C)** During the interval of day 4 to day 7 post insult a Pearson correlation coefficient value of |*ρ*| > 0.9 was deemed high (N=5-12).

Having knowledge of the extent and nature of the correlation among variables in a study is crucial from predictive and causative perspectives. Discovering the correlation among immune cells at various stages of stroke is particularly valuable since it might not be possible to measure the dynamics of all cell types during an experiment. We consider the spleen instead of blood as the peripheral source of leukocytes because the spleen is a primary source of infiltrated immune cells to the brain after stroke. Although immune cells may traffic into the brain through blood circulation, they may also migrate *via* other means such as lymphatic vessels; thus focusing on blood may not reflect the immune cells migration from the periphery to the CNS. Furthermore, cell counts can be attained from the spleen but not from blood since it is not feasible to measure the entire population of immune cells from blood. This is because it is not possible to attain the blood volume from each animal since the blood level is reduced following stroke with individual variability. For each time point after stroke, the mean of every measured cell type was computed across the mice in order to attain an aggregate account across the population. Subsequently, Pearson correlation coefficients were computed over different intervals of time post infarct – this included day 0-7, and its constituent intervals of day 0-3 and day 4-7. This information is contained in the heatmaps in **Supplementary Figure 2** while the correlation levels that have been deemed as significant are shown in **Figure 7**. In providing a graphical depiction of the discovered correlation structure in **Figure 7**, the relationships between cells in a sham spleen with the other post-stroke cell types are not likely to be informative and thus were not included in this figure.

**Figure 7A** recapitulates the significant correlations measured over the 7-day interval following the insult. The majority (i.e. 4/6) of high positive correlations among the cells in the brain exist among cell types associated between the ipsilateral and contralateral hemispheres. In particular, the number of B and T cells that are present in the two hemispheres share high positive correlation. The number of microglia in the ipsilateral hemisphere is highly correlated with the number of B cells on the contralateral side. The number of neutrophils in the ipsilateral side shares high positive correlation with the number of macrophages in the contralateral side. Within the ipsilateral side of the brain, there is high positive correlation among the number of microglia and B cells, as well as among the number of neutrophils and macrophages. The only high positive correlation noted within the cells in the spleen is between the number of macrophages and B cells in a stroke spleen, and the number of T and B cells in a stroke spleen. It is intriguing that when considering the 7-day post-insult interval in its entirety, high correlations do not exist between any cell type measured in the brain and the stroke spleen. It is also interesting that no significant negative correlation exists among the cells in the two brain hemispheres and the stroke spleen.

The above analysis was repeated with the Pearson correlation coefficients computed separately for data spanning the two temporally-distinct intervals of 0-3 days and 4-7 days post-infarct. We note from **Figures 7B, C** that there exists a significantly greater amount of positive and negative correlations among the number of the various cell types present than when we considered the aggregate 7-day period. It is striking that the correlations were apparently weakened by considering a larger temporal window of 7 days across the same mice. In fact, for the 4-7 day interval, much higher correlation existed among the various cell types which led us to adjust the high-negative and high-positive thresholds to -0.9 and 0.9, respectively. There is a 100% increase (8 to 16) in comparing the number of relations for 0-3 days to those for the 7-day period (both |*ρ*| > 0.8). Also, there is a 212.5% increase (8 to 25) in comparing the number of relations for 4-7 days to those for the 7-day period (both |*ρ*| > 0.9). For the 0-3 day post-infarct interval **Figure 7B** indicates that in the brain, the majority (i.e. 5/7) of high positive correlations exist among the cell types across the ipsilateral and contralateral hemispheres rather than within each hemisphere. The number of microglia in the ipsilateral and contralateral hemispheres share a high degree of positive correlation. It is noted that the number of T cells in the contralateral hemisphere are positively correlated with the number of macrophages in both the ipsilateral and contralateral hemispheres. The number of macrophage, T cells, and B cells in the stroke spleen share only high negative correlations with cells in the brain. More specifically, high negative correlation is noted between the macrophages in the ipsilateral hemisphere and the stroke spleen. This negative correlation suggests that the splenic macrophages infiltrate the brain after stroke, which is consistent with previous findings (19–22). Also, high negative correlation is noted between the number of B cells in the contralateral hemisphere and the stroke spleen. In a stroke spleen, the number of T cells has high positive correlation with the number of macrophages as well as the number of B cells. Within the stroke spleen, the number of macrophages has high positive correlation with the number of B cells present. There are other relationships that can be gleaned from **Figure 7** and are not explicitly mentioned in the interest of space.

The relationship among the cell types during the 4-7 day post-insult interval is illustrated in **Figure 7C**. The number of microglia in the ipsilateral hemisphere is negatively correlated with several other cell types in the same hemisphere including neutrophils, T cells, and macrophages. In the contralateral hemisphere the number of T cells has a high positive correlation with the number of T cells, macrophages, and neutrophils present in the ipsilateral hemisphere. Furthermore, there is high positive correlation among B cell numbers in the ipsilateral and contralateral hemispheres. In the spleen, high positive correlation is evident between the number of T cells and B cells, the number of B cells and neutrophils, as well as the number of neutrophils and macrophages. Interestingly, there is no high negative correlation among the different cell types accumulating within the spleen – this was also noted for the 0-3 days analysis. When comparing the correlation between the different cell types present in the brain and the spleen, the number of macrophages in the ipsilateral hemisphere has a high negative correlation with the number of B and T cells in the stroke spleen. Similarly, the neutrophils in the contralateral hemisphere has a high negative correlation with the number of B and T cells in the spleen. Conversely, the number of microglia in the contralateral hemisphere has a high positive correlation with the macrophages and neutrophils in the spleen. We remark that while the data and analysis that is reflected in **Figure 7** and **Supplementary Figures 2** does not consider cells migrating from the spleen to the brain *via* cell tracking experiments, it does provide correlational evidence. For instance, **Figure 7B** shows that during the 0-3 day post insult interval negative correlation exists between the number of macrophages in the spleen and the ipsilateral hemisphere. During the same temporal interval negative correlation is also noted between the number of B cells in spleen and the contralateral hemisphere. The correlational findings are supportive of the cells’ migration from the spleen to the brain following the insult.

### Recruited Myeloid Cells Worsen Stroke Outcome While Microglia and Splenic Adaptive Immune Cells Improve Stroke Outcome

**Figure 8:**
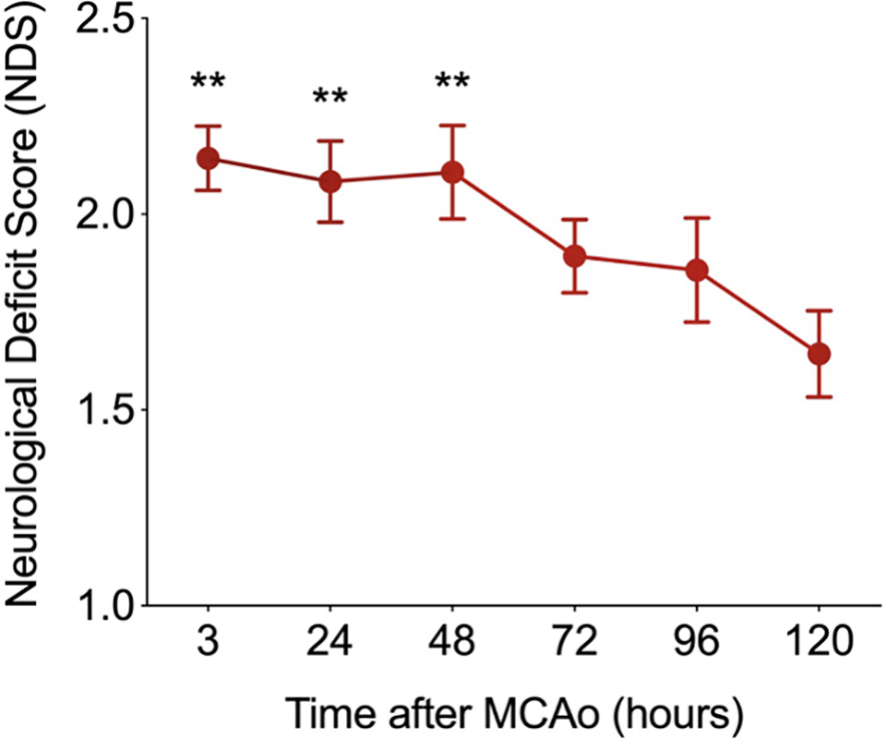
The relationship between the neurological deficit score (NDS) and the time after MCAO (N=14). The improvement in NDS with time was significant (i.e. **p < 0.01) when comparing the final time point recorded at t=120 hours to the first three recorded time points after MCAO. A two-sample t-test was used to assess significance.

**Figure 9:**
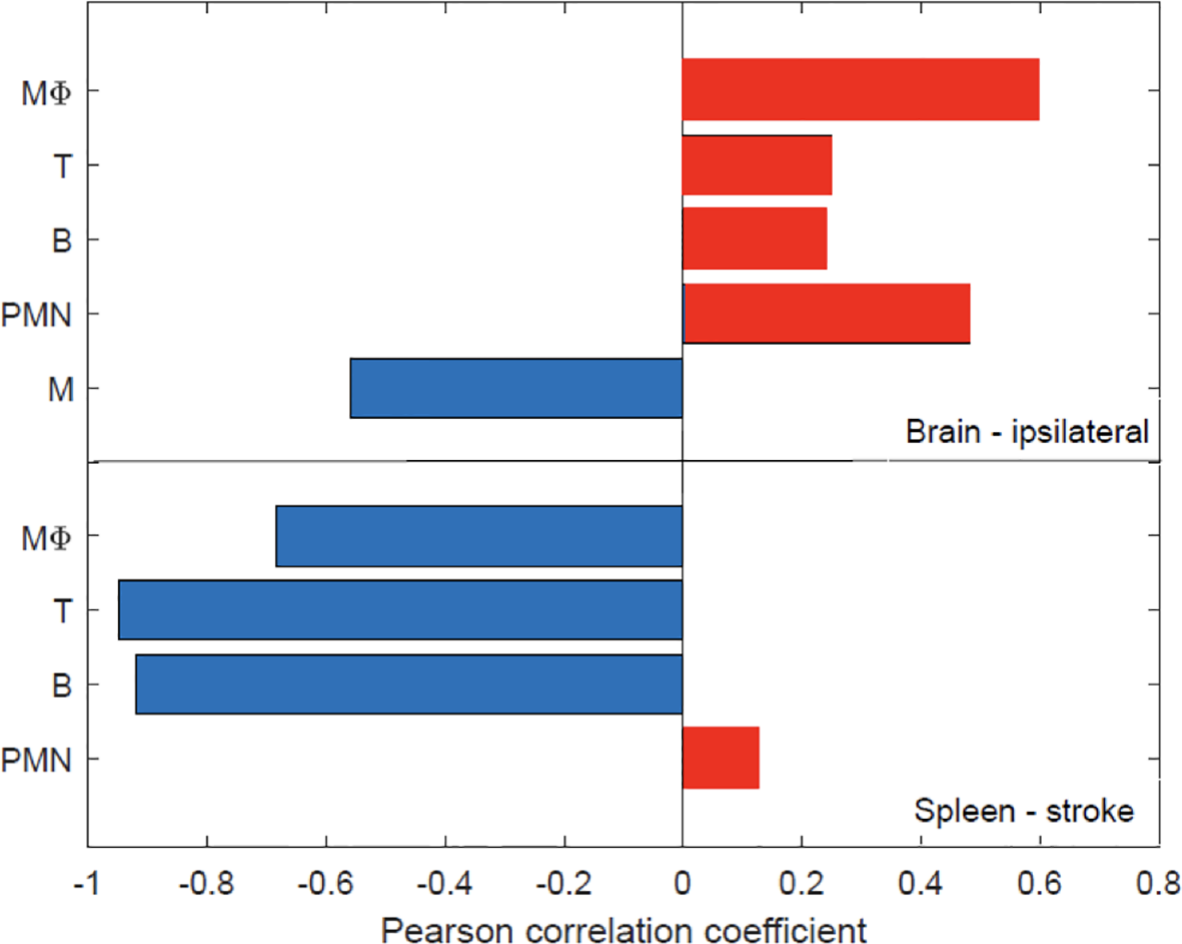
The relationship between the number of immune cells present in the brain and spleen and stroke outcome as determined *via* the neurological deficit score (NOS). A Pearson correlation coefficient was computed among the NDS score and the number of cells in the brain and spleen after stroke - the red and blue colors denote positive and negative correlation, respectively. The number of cells were recorded directly after MCAO (i.e. 0 hours) as well as at 24, 48, 72, 96, 120 hours post-MCAO. Similarly, the NDS was measured at perfusion (i.e. 0 hours) and 24, 48, 72, 96, 120 hours following perfusion. N=14 mice were used, but recordings of each cell type at every time point was not possible across all animals **Supplementary Table 1** for the number of various cell type measurements that were attained at each time point.

The relationship between the NDS and the time after MCAO is shown in **Figure 8** with an improvement in stroke outcome being significant only when comparing the final time point (t=120 hours) to the first three recorded time points. The Pearson correlation coefficient was computed among the NDS and the cell types discussed so far in the brain and spleen of the mice (**Figure 9**). In the brain we note a negative correlation of NDS with the number of microglia present in either hemisphere. The NDS shows weak positive correlation with numbers of neutrophils, T cells, and B cells (max ρ=0.48 for the neutrophils in the ipsilateral hemisphere) and positive correlation with the number of macrophages in the ipsilateral hemisphere (ρ=0.59) but is not strongly correlated with the number present in the contralateral hemisphere (ρ=0.47). In the spleen of stroke mice there is a high negative correlation between the NDS and the numbers of T cells, B cells and macrophages, but the NDS is uncorrelated with the number of neutrophils when considering the first five days after MCAO. However, as shown in **Figure 9**, the number of neutrophils present in the spleen positively correlates with NDS in the sham control condition. The number of B cells and macrophages also exhibits positive correlation with NDS in the sham spleen, while the number of T cells shows a negative correlation. In addition, the correlation between the number of cells present and the NDS is more similar across the two brain hemispheres than between the stroke and sham spleens. A decrease in cells in the spleen is linked to a worse stroke outcome as measured by the NDS. As shown in **Figure 9**, a negative correlation between splenic macrophage, T, and B cells and the NDS suggests that these splenic immune cells may aid in stroke recovery. This is an instance of investigating the dynamics of specific immune cells that contribute to cerebral injury. While such analysis has been conducted in a number of works, a consensus does not seem to exist. For instance, it has been noted that modulating macrophage response does not influence short-term or longer-term neural recovery (26), and that the total number of spleen-derived macrophages does not significantly contribute to acute infarct development (27). The factors that drive the time-dependent fluctuations of macrophage phenotypes following cerebral ischemia have still yet to be completely identified (28). During the first 5 days post stroke, positive correlation between the infiltrated macrophages and neutrophils with the NDS suggests a detrimental effect of infiltrating myeloid cells on stroke severity, consistent with the correlation analysis performed in (9). The observation that the number of microglia in the ipsilateral hemisphere exhibits negative correlation with NDS implies a protective mechanism offered by microglia against stroke severity.

## Discussion

A dearth of computation currently exists to characterize the inflammatory processes associated with cerebral ischemia. This work presents analysis to quantify the accumulation of immune cells following MCAO-induced cerebral ischemia in mice. By developing a model to quantify the biological occurrences, we hope to have motivated a means to study the cellular dynamics associated with cerebral ischemia. For instance, while there has been no concrete conclusion on the function of peripheral myeloid cells and microglia post stroke, the presented correlational analysis suggests that microglia may have a protective effect while infiltrating peripheral myeloid cells likely exert detrimental effects at an early stage post stroke (day 0 to 5 as shown in **Figure 9**). Although it remains unknown whether activated microglia are beneficial or detrimental to stroke recovery, increasing evidence aligns with our findings that microglia exhibit beneficial effects during the first 5 to 7 days post ischemic stroke. In particular, microglia exhibited a dominant neuroprotective M2-like response (phagocytosis and tissue repair) for the first seven days post stroke, characterized by increased expression levels of CCL22, IL10, CD206, arginase-1 and TFG-β (13, 29–32). The significance of the finding rests in the number of the various cell types in the brain following the insult being a biomarker of the outcome. Furthermore, if the dynamics of one cell type is found to be reflective of the dynamics of other cell types, such correlational structure can be harnessed to reduce the amount of measurements, analysis, and processing necessary to model or predict the dynamics of the system. The correlation among the various cell types in the contralateral and ipsilateral hemispheres as well as the spleen were noted as displaying a time-varying structure and being quite different among the three temporal windows that were considered. This is important for investigating the role of spleen in the post-stroke response. It has been suggested that the spleen responds in a similar manner in stroke subjects as in animal models (33). Furthermore, studies have discussed that the immune cells from the spleen travel to the brain to exacerbate neural injury and hence a reducing of peripheral inflammation is beneficial for mitigating injury (21, 34). The prior studies, however, had not examined the correlation among immune cells released by the spleen with cells in the brain for different intervals following stroke.

While we have considered the NDS as a measure of stroke outcome in this study, other outcome measures of neural plasticity can also be used (17). In fact, the consideration of cell correlates of stroke-related cognitive impairment are an important future consideration. Examination of synaptic plasticity and how it is affected by infarct severity and the post-stroke inflammatory response are intriguing future avenues to pursue from a quantitative perspective. This is particularly important when considering that neurotoxic effects from post-stroke pro-inflammatory responses may interact with inflammation-associated neuroprotection and nerve regeneration (35). Such studies would require the consideration of neurons into the prospective model. The conducted experiments focused on the cellular response in the brain and spleen. Since there are virtually no peripheral immune cells in the brain after 7 days following MCAO, it was necessary to sacrifice the animals at 7 days thus precluding a scrutiny of learning and memory that are typically evaluated at least two weeks following MCAO. Thus, we believe the NDS was sufficient to represent the level of brain injury due to focal stroke during the 7 days after stroke onset. It is also noteworthy that the NDS is a good indicator of stroke outcome when considering a relatively short time interval following stroke in mice – i.e. 7 days (36). Works such as (32) have considered monitoring of animals up to 28 days following MCAO, thus having an opportunity to perform a battery of behavioral tests. Assessing the performance in such tests over the longer time interval is a future avenue that will be considered.

It is important to be cognizant of the sources of noise that arise in the induction of stroke and the measurement of cells in the brain and spleen. It is equally important to be aware of biological variability in the subjects. The former presents disturbances that reflect the limits in measurement and experimental procedures while the latter reflect somewhat unavoidable perturbations that are inherent to a subject. Prior to discussing the prospective modeling of such randomness it is necessary to identify sources of variability. The following factors may be considered as stochastic processes that perturb the aforementioned variables X(t), Y(t), D(t), L_Bc_(t), L_Tc_(t), and Z(t). The temperature during an experiment is frequently not constant across different studies. Increased temperature is detrimental to the survival of neurons and may cause damage to all of the mentioned cell types. In fact, higher than normal temperatures contribute to a larger infarct volume while hypothermia has been shown to reduce the pathology associated with stroke (37–40). It should be evident that this is not a measurement-related perturbation, but rather an environmental variability that will affect the outcome. The degree of collateral circulation is also a source of variability. Collateral circulation can reduce the decrease in blood flow within the brain parenchyma during the occlusion and may also alter reperfusion, affecting both neuronal survival and numbers of immune cells that reach the brain parenchyma. The degree of collateral circulation is a known contributor to the biological variance that results in different stroke outcomes across different strains of mice.

The results communicated in **Figures 4**, **5**, **7**–**9** encompass novel data and analysis attained *via* a combination of induced MCAO, flow cytometry, neurological deficit scoring, and statistical analysis. Several advancements are necessary for the presented work to be part of a large effort to quantify and predict cellular dynamics associated with cerebral ischemia and outcome. An intriguing study would entail correlating the infarct volume of a subject with the rates of cell accumulation in the ischemic brain. As additional data becomes available it will be possible to incorporate additional variables. For instance, the location of the infarct in the brain is a crucial determinant for the loss of motor function. A relatively small infarct volume centered in the motor cortex would be expected to cause a devastating loss of motor function. This is in contrast to white matter/lacunar strokes that can show faster recovery time in comparison to a gray matter large vessel stroke (41). Another relevant factor is the presence of co-morbidities such as diabetes and hypertension. At the cellular level, an additional consideration is the role of neurons and astrocytes in stroke outcome. Astrocytes protect uninjured parts of the brain by walling off the stroke area via the formation of a glial scar that reduces exposure of viable brain tissue to toxic inflammatory mediators (42, 43). 

In summary, the quantitative analysis of cell dynamics and the correlational study presented serve to further elucidate the interactions among various cell types following stroke. While spleen weights and morphometrics following stroke has been reported in prior works such as (18), splenic cell dynamics have been presented here. To the best of our knowledge the results in this work are the first to investigate the dynamics of spleen cells after stroke in conjunction with cells in the brain. The work is novel in revealing the migration of splenic macrophages, neutrophils, T cells, and B cells into the ipsilateral and contralateral hemispheres of a post-stroke brain *via* quantitative analysis. We have found that the infiltrated macrophages in the ischemic hemisphere positively correlate with neutrophils. This implies their synergic effect in migrating into the brain after stroke. It was also observed that during infiltration of adaptive immune cells, the number of neutrophils correlate positively with T cells suggesting that neutrophils contribute to T cell infiltration following stroke. This was also supported by observation in the ipsilateral brain as large changes in the number of neutrophils occurred earlier than that in T cells. The correlational analysis supports the concept that microglia have a protective effect while infiltrating peripheral myeloid cells exert detrimental effects in stroke outcome. Although the conclusions are based on correlational rather than casual evidence, they constitute a quantitative basis for understanding the brain and spleen immune cell dynamics following cerebral ischemia.

## Data Availability Statement

The original contributions presented in the study are included in the article/**Supplementary Material**. Further inquiries can be directed to the corresponding authors.

## Ethics Statement

The animal study was reviewed and approved by Stanford Institutional Care and Use Committees.

## Author Contributions

SS analyzed the data and wrote the manuscript. QL designed the experiments, interpreted the data, and wrote the manuscript. All authors contributed to the article and approved the submitted version.

## Conflict of Interest

The authors declare that the research was conducted in the absence of any commercial or financial relationships that could be construed as a potential conflict of interest.
