# Supplementary material for: Quantitative and Correlational Analysis of Brain and Spleen Immune Cellular Responses Following Cerebral Ischemia
Source: Front Immunol. 2021 Jun 8;12:617032. doi: 10.3389/fimmu.2021.617032 (PMC8238006; doi:10.3389/fimmu.2021.617032)
Supplement: Supplementary file 1 [file Table_1.docx]

|  | 0 days | 1 day | 2 days | 3 days | 4 days | 5 days | 6 days | 7 days |
| --- | --- | --- | --- | --- | --- | --- | --- | --- |
| MΦ, ipsilateral | 6 | 7 | 7 | 7 | 7 | NA | 7 | 6 |
| T, ipsilateral | 6 | 8 | 8 | 10 | 7 | NA | 10 | 6 |
| B, ipsilateral | 6 | 8 | 10 | 10 | 7 | NA | 8 | 6 |
| PMN, ipsilateral | 8 | 8 | 7 | 8 | 7 | NA | 7 | 6 |
| M, ipsilateral | 8 | 8 | 7 | 9 | 7 | NA | 8 | 8 |
| MΦ, contralateral | 6 | 7 | 7 | 7 | 7 | NA | 7 | 6 |
| T, contralateral | 6 | 8 | 8 | 10 | 7 | NA | 10 | 6 |
| B, contralateral | 6 | 8 | 10 | 10 | 7 | NA | 8 | 6 |
| PMN, contralateral | 8 | 8 | 7 | 8 | 7 | NA | 7 | 6 |
| M, contralateral | 8 | 8 | 7 | 9 | 7 | NA | 8 | 8 |
| MΦ, spleen-stroke | 8 | 7 | 10 | 12 | 9 | NA | 7 | 8 |
| T, spleen-stroke | 6 | 7 | 9 | 10 | 7 | NA | 7 | 10 |
| B, spleen-stroke | 6 | 7 | 14 | 13 | 7 | NA | 7 | 8 |
| PMN, spleen-stroke | 10 | 7 | 12 | 12 | 12 | NA | 7 | 8 |
| MΦ, spleen-sham | 8 | 8 | 5 | 5 | 6 | NA | 6 | 7 |
| T, spleen-sham | 6 | 8 | 5 | 5 | 6 | NA | 6 | 5 |
| B, spleen-sham | 6 | 8 | 6 | 5 | 6 | NA | 5 | 6 |
| PMN, spleen-sham | 10 | 8 | 5 | 5 | 6 | NA | 6 | 6 |
| NDS | NA | 14 | 14 | 14 | 14 | 14 | NA | NA |

Supplementary Table 1: An itemization of the number of mice that underwent MCAO and had of cell types counted and NDS measured at the considered time points in the presented data. The above N values were used to arrive at the numerical results – i.e. mean cell counts, correlation levels, and mean NDS values. The different number of samples across days is because outlier removal was performed when examining the number of the various cell types measured over the days for each animal.
