# Supplementary figures and images for: Quantitative and Correlational Analysis of Brain and Spleen Immune Cellular Responses Following Cerebral Ischemia
Source: Front Immunol. 2021 Jun 8;12:617032. doi: 10.3389/fimmu.2021.617032 (PMC8238006; doi:10.3389/fimmu.2021.617032)

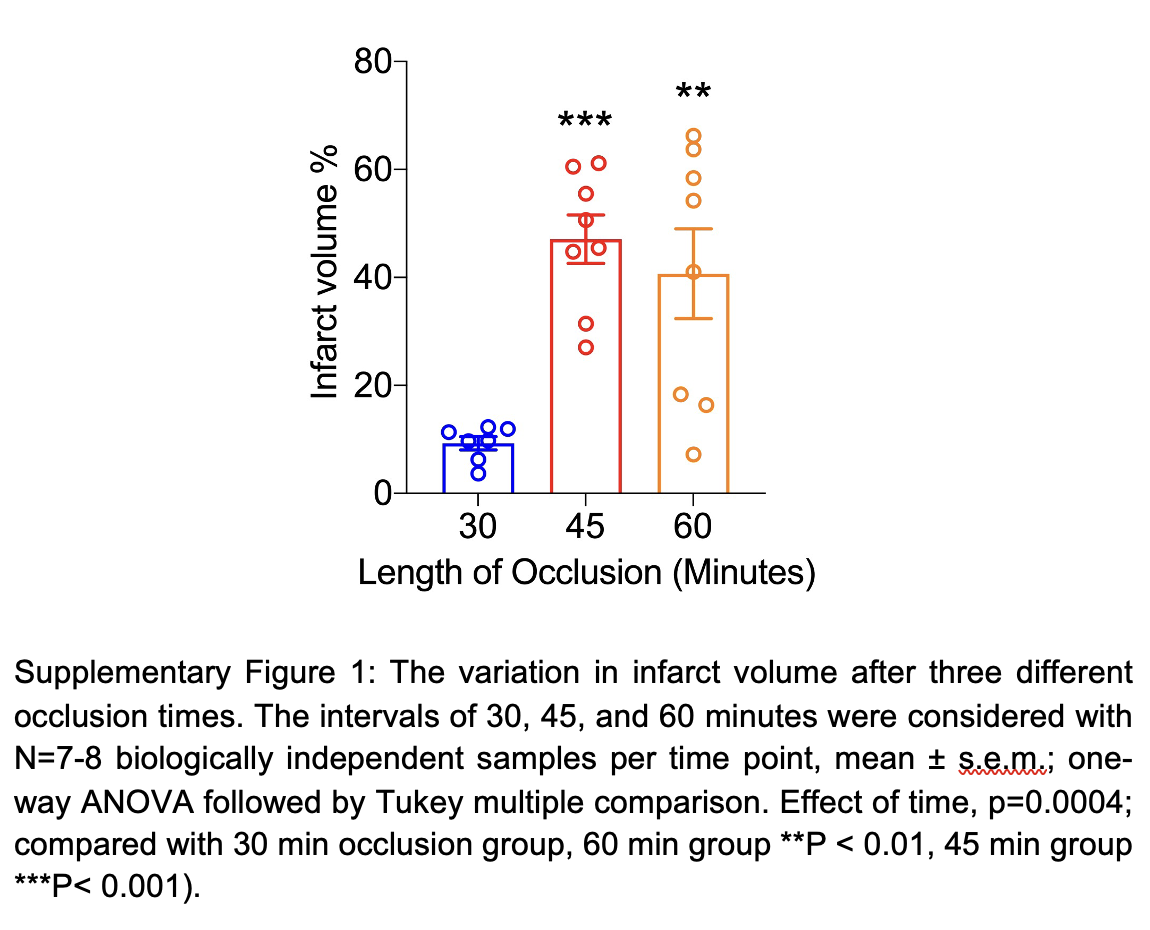

Supplement: Supplementary file 2 [file Image_1.tiff]

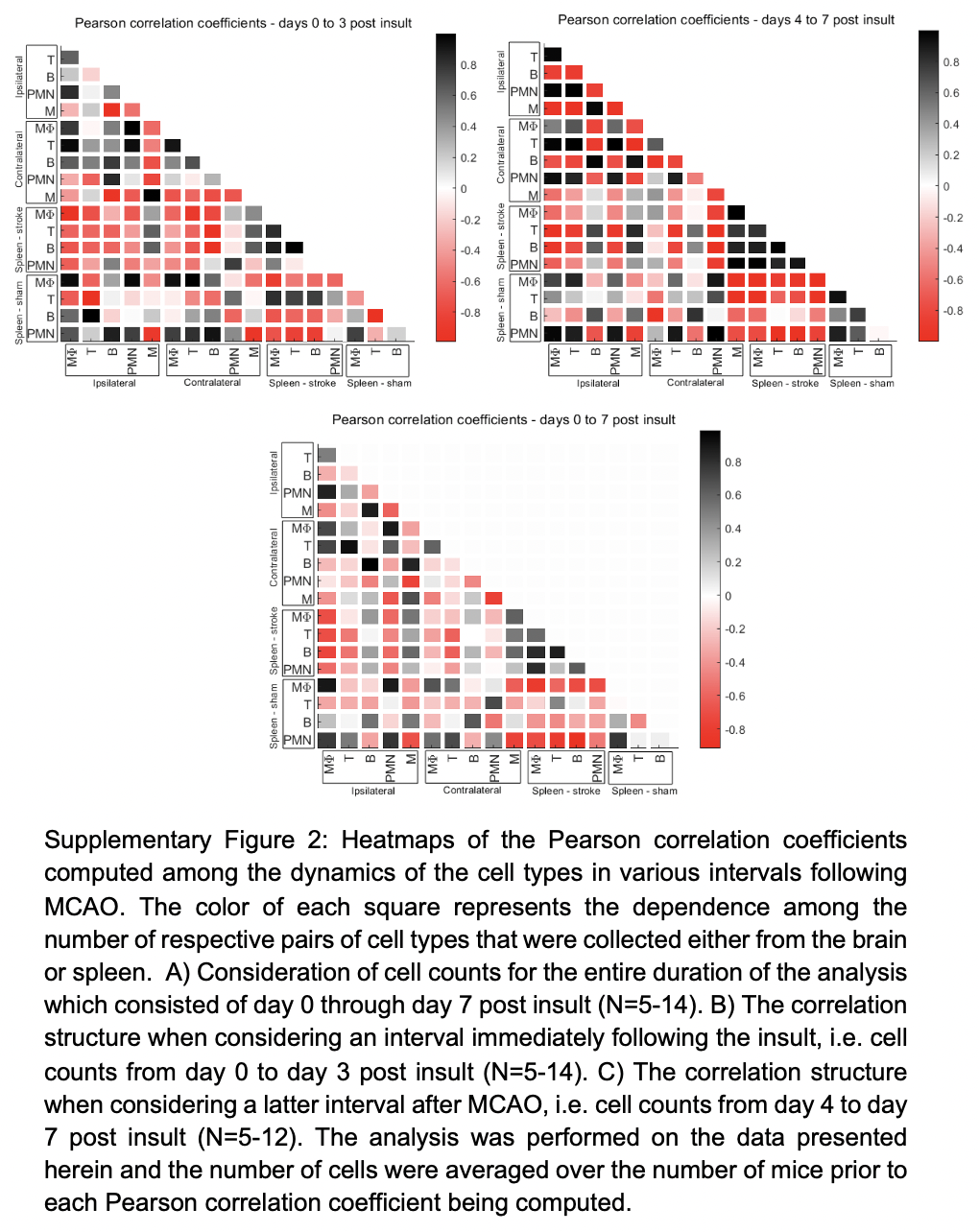

Supplement: Supplementary file 3 [file Image_2.tiff]
